# Comparative Analysis of Kidney and Simultaneous Pancreas–Kidney Transplantation: Long-Term Outcomes in Type 1 Diabetic Patients with End-Stage Kidney Disease

**DOI:** 10.3390/jcm15072565

**Published:** 2026-03-27

**Authors:** Jacek Ziaja, Monika Widera, Aureliusz Kolonko, Aleksander J. Owczarek, Dorota Kamińska, Robert Świder, Agata Góral, Sylwia Sekta, Robert Król, Jarosław Czerwiński, Magdalena Durlik, Andrzej Więcek

**Affiliations:** 1 Department of General, Vascular and Transplant Surgery, Medical University of Silesia, 40-055 Katowice, Poland; 2 Department of Nephrology, Transplantation and Internal Medicine, Medical University of Silesia, 40-055 Katowice, Poland; 3 Health Promotion and Obesity Management Unit, Department of Pathophysiology, Medical University of Silesia, 40-055 Katowice, Poland; 4 Department of Non-Procedural Clinical Sciences, Faculty of Medicine, Wroclaw University of Science and Technology, 50-370 Wroclaw, Poland; 5 Department of Transplantology, Immunology, Nephrology and Internal Medicine, Medical University of Warsaw, 02-091 Warsaw, Poland; 6 Faculty of Medicine, Wroclaw Medical University, 50-367 Wroclaw, Poland; 7 Polish Transplant Coordinating Center Poltransplant, 02-001 Warsaw, Poland; 8 Department of Emergency Medicine, Medical University of Warsaw, 02-091 Warsaw, Poland

**Keywords:** type 1 diabetes, end-stage kidney disease, kidney transplantation, simultaneous pancreas–kidney transplantation, long-term results

## Abstract

**Background**: The primary aim of pancreas transplantation in type 1 diabetic kidney transplant recipients is to reduce mortality caused by complications of progressing cardiovascular diseases and to protect kidney graft from the recurrence of diabetic nephropathy. The aim of this study was to analyze the results of the first deceased donor kidney transplantation (KTx) in patients with end-stage kidney disease caused by long-lasting type 1 diabetes (T1D), performed alone or simultaneously with the pancreas (SPK), in a long-term follow-up period. **Methods**: Groups of 101 consecutive T1D patients after KTx and 93 patients after SPK performed in two transplant centers with a minimal follow-up period of 5 years were included in the analysis. **Results**: Recipient, kidney graft, and death-censored kidney graft survival in a follow-up period of up to 20 years did not differ between KTx and SPK groups. In the entire observation period, total diabetes duration was shorter in the SPK group compared to KTx (28.1 ± 7.4 vs. 34.9 ± 11.0 years), and myocardial infarction (21 vs. 7%), limb amputation (12 vs. 3%), and proteinuria (40 vs. 18%) were more common in the KTx group than in SPK. The estimated glomerular filtration rate was lower in KTx recipients compared to SPK. Recipient survival was affected by the recipient’s age and the duration of dialysis vintage, and kidney graft survival was affected by the duration of dialysis vintage, episodes of acute rejection, and high pretransplant sensitization in patients. **Conclusions**: Despite reduced diabetes duration, a decreased frequency of cardiovascular disease complications and better kidney graft function, a simultaneously transplanted pancreas does not improve patient or kidney graft survival in T1D kidney recipients.

## 1. Introduction

Kidney transplantation (KTx) has a beneficial impact on the survival of patients with type 1 diabetes (T1D) who develop end-stage kidney disease (ESKD) [1]. Kidney transplantation may be complemented in these patients by the transplantation of a pancreas procured from a deceased donor, performed either simultaneously with a kidney procured from the same donor, or later, after the transplantation of a kidney procured from a living or deceased donor [2,3].

The primary goals of dual-organ transplantation are to slow the progression of cardiovascular diseases (CVDs) and reduce mortality caused by CVD complications. Additionally, it aims to protect the transplanted kidney from the recurrence of diabetic nephropathy [4,5]. Indeed, many studies from the first decade of the 21st century confirmed higher survival rates of recipients and kidney graft in patients after simultaneous pancreas–kidney transplantation (SPK) or pancreas after kidney transplantation compared to patients who underwent only the transplantation of a kidney procured from a living or deceased donor in a long-term follow-up period [2,3,6].

On the other hand, some reports suggest that with regard to the survival rate among SPK recipients, the advantage of pancreas transplantation is small compared with the benefits associated with better health status of the recipient receiving a kidney from a younger and healthier donor, or the benefits of earlier transplantation [7].

Notably, most of the cited analyses comparing therapeutic options for patients with ESKD and T1D were conducted in the United States. Given differences in the long-term results of KTx in the United States and Europe, it is reasonable to expect that the results of transplantation, for both SPK and KTx alone, may also differ in T1D recipients [8]. Furthermore, discrepancies in the benefits of additional pancreas transplantation in T1D kidney recipients have been observed in recent studies from Japan and New Zealand [9,10].

Additionally, improved survival has been observed in T1D patients with ESKD undergoing both dialysis treatment and KTx, which may influence the outcomes of comparative analyses assessing the efficacy of SPK and KTx alone in these patients [1].

To date, no studies in Poland have compared the outcomes of SPK and KTx in terms of recipient and kidney graft survival, particularly during long-term follow-up. Our preliminary report revealed that during a 7-year follow-up period, pancreas transplantation reduced cardiovascular risk and prevented the development of proteinuria but did not improve patient and kidney graft survival in T1D recipients [11].

Given the continuous progress in immunosuppressive treatment, surgical techniques, and perioperative management, as well as the considerations outlined above, we performed a study aimed at analyzing long-term patient and graft survival, kidney graft function and cardiovascular complications following the first deceased donor kidney transplantation in patients with end-stage kidney disease caused by type 1 diabetes mellitus performed alone or simultaneously with pancreas transplantation.

## 2. Materials and Methods

A total of 201 adult patients with T1D and ESKD (aged 18 years and older) underwent KTx at two transplant centers in Upper and Lower Silesia, Poland, between 1998 and 2019: 143 in Katowice and 58 in Wrocław, respectively.

In four cases, recipients underwent kidney retransplantation (following KTx or SPK performed at another transplant center), and in three cases, patients received organs from living donors; therefore, they were excluded from further analysis.

Among the remaining 194 patients included in the study after the first KTx, 93 patients (47.7%) received a simultaneous pancreatic graft from the same donor (all at the transplant center in Katowice, SPK group). The remaining 101 patients constituted the kidney-only group treated with insulin (KTx group).

As the aim of the study was to analyze the long-term outcomes following the first donor kidney transplantation, patients who experienced graft loss and subsequently underwent kidney retransplantation during the follow-up period were not excluded from the study. However, parameters related to second kidney graft (graft survival and kidney function) were not included in the analysis of kidney graft outcomes.

In all patients, T1D was diagnosed in childhood or adolescence, insulin therapy was required from the onset of the disease, and T1D was the primary cause of ESKD necessitating dialysis. Due to the lack of unequivocal criteria for qualifying patients with ESKD in the course of T1D for dual-organ transplantation in Poland, the decision on the type of transplant (SPK or KTx) was based on clinical parameters of potential recipients: younger patients with lower BMI and without severe cardiac comorbidities were eligible for SPK, whereas those not meeting these criteria underwent KTx alone. Close collaboration and geographic proximity between the participating centers allowed for comparable access to SPK for patients qualified at both transplant centers. Patients qualified for transplantation were given the option to choose between SPK or KTx, and if a deceased kidney-only donor was available, recipients awaiting SPK transplantation could decide to undergo KTx.

All recipients were listed on the National Waiting List maintained by the Polish Transplant Coordinating Centre Poltransplant, Warsaw (Poltransplant), and potential SPK recipients were given priority to obtain organs regardless of HLA mismatch.

All organs were procured from deceased donors after brain death. In all patients, the kidney graft was placed extraperitoneally and anastomosed to the external iliac artery and vein. In SPK recipients, pancreatic graft was placed intraperitoneally with enteric drainage of pancreatic juice and systemic drainage of venous blood [12].

The immunosuppressive protocol applied in both centers was based on national Recommendations Concerning Immunosuppressive Treatment after Vascularized Organs Transplantation [13,14].

Data were obtained from a registry of all patients undergoing KTx at the transplant center in Katowice, in which information on operated-on patients is collected on an ongoing basis, including long-term follow-up data. Additionally, the analysis relied on outpatient records of patients who had undergone KTx at the transplant centers in Wrocław and Warsaw, as well as on data obtained from Poltransplant.

The minimum follow-up period was 5 years, and the maximum was 20 years. The end of follow-up was defined as the last outpatient visit, telephone contact, or patient death confirmed by the Poltransplant registry.

The retrospective analysis included comparisons between SPK and KTx groups regarding recipients, donors, and transplantation procedure parameters, as well as applied immunosuppression (Table 1). In a comparison of kidney function directly after transplantation, early graft function was defined as immediate if serum creatinine concentration on the 3rd post-transplant day was ≤264 µmol/L, slow if serum creatinine concentration on the 3rd post-transplant day was >264 µmol/L, and delayed if dialysis therapy was required during the first week after transplantation.

In the main part of the study, the following parameters were compared between the study groups: the frequency of cardiovascular comorbidities, total duration of dialysis vintage and diabetes, and incidence of kidney retransplantation (Table 2), as well as patient and first kidney graft survival (Table S1) and kidney graft function (Table S2), assessed at 5-year intervals. 

The assessment of kidney graft function (first graft) was evaluated based on the estimated glomerular filtration rate value (eGFR) calculated according to the Modification of Diet in Renal Disease (MDRD) formula.

A cardiovascular event was defined as myocardial infarction, stroke, or transient ischemic attack (TIA). The occurrence of proteinuria during the entire follow-up period was also assessed and defined as persistent albumin excretion exceeding 300 mg/dL.

The total duration of T1D was calculated as the duration prior to transplantation plus the follow-up period for the KTx group, and as the duration of diabetes prior to transplantation plus the follow-up period minus the period of functioning pancreatic graft in the SPK group (years). The total duration of dialysis was defined as duration of dialysis prior to transplantation plus the time after kidney graft loss minus the period with retransplanted kidney (months).

Pancreatic graft survival was assessed at 5-year intervals (Table S1).

**Figure 1:**
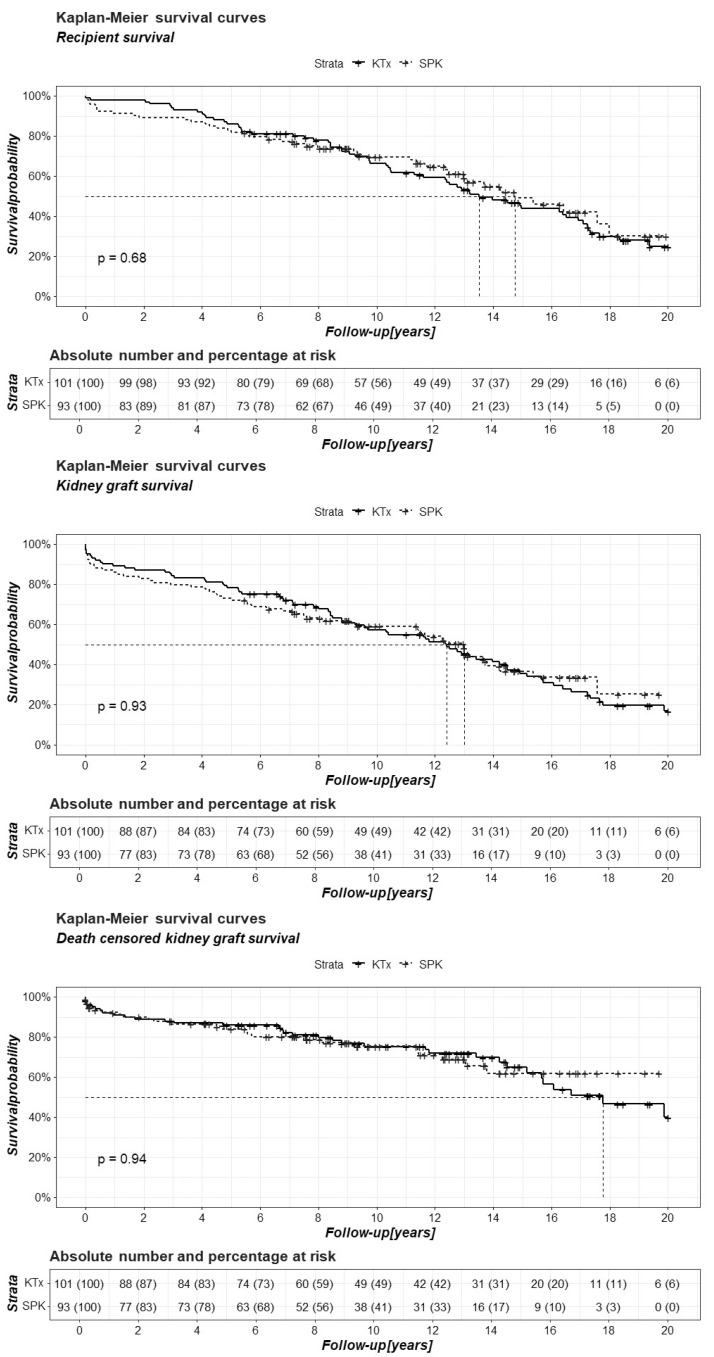
Comparison of recipient, kidney graft, and death-censored kidney graft survival between patients after kidney transplantation alone (KTx) and simultaneous pancreas–kidney transplantation (SPK) in a 20-year follow-up period.

**Figure 2:**
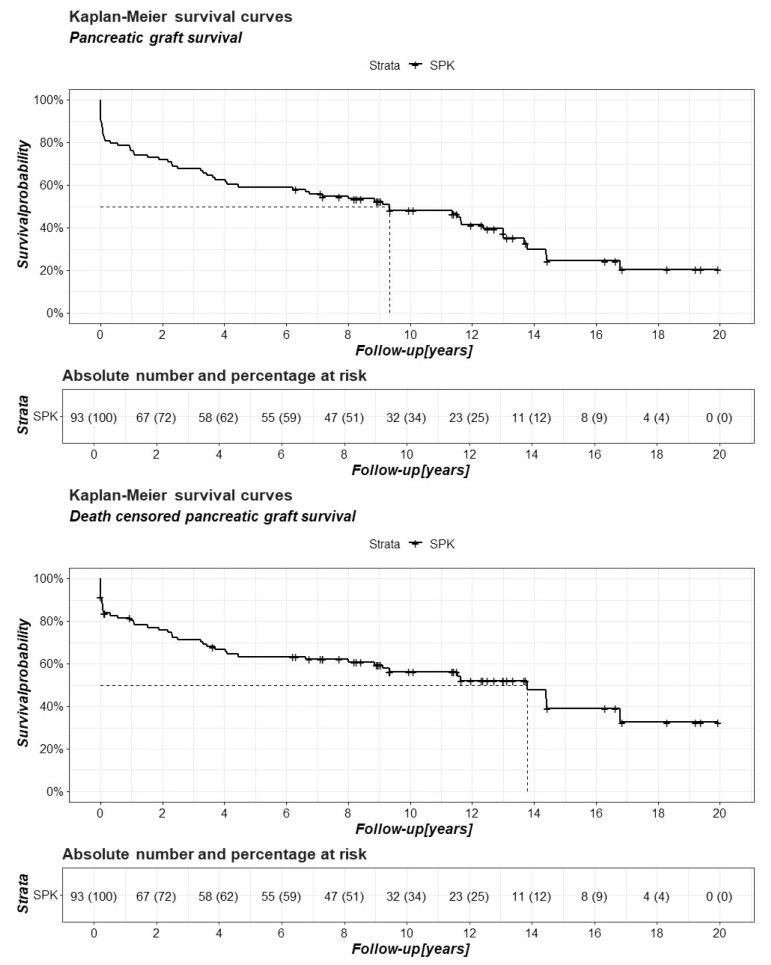
Pancreatic graft and death-censored pancreatic graft survival in patients after simultaneous pancreas–kidney transplantation (SPK) in a 20-year follow-up period.

Recipient survival, kidney graft survival, and death-censored kidney graft survival were also compared between the analyzed groups using Kaplan–Meier curves. Pancreatic graft survival and death-censored pancreatic graft survival were also analyzed using this method (Figure 1 and Figure 2).

Finally, in the overall study population, an attempt was made to identify factors influencing patient and kidney graft survival. Donor-related (age, gender, BMI and trauma as the cause of death), recipient-related (age, gender, BMI, duration of diabetes and dialysis therapy prior to transplantation, previous cardiovascular events [myocardial infarction, stroke or TIA], and high sensitization [PRA > 20%]), and transplantation-related (SPK or KTx, mismatch HLA class I or II, kidney cold ischemia time [CIT], slow or delayed kidney graft function, induction therapy, and episodes of acute rejection) factors were included as potential predictors of patient and kidney graft survival (Table 3 and Table S3).

### Statistical Analysis

Statistical analysis was performed using STATISTICA 13.0 PL (Tibco Software Inc., Palo Alto, CA, USA) and R software (v 4.4.0; R Development Core Team [2008]; R: A language and environment for statistical computing. R Foundation for Statistical Computing, Vienna, Austria). Statistical significance was set at a *p*-value below 0.05. All tests were two-tailed. 

Missing data were assumed to be missing at random (MAR) with a maximum missingness of 9.8%, except for clinical data related to cardiovascular diseases and amputation after transplantation, where missingness reached up to 18.6%. Imputations for missing data were not performed. Nominal and ordinal data are presented as percentages, whereas continuous variables are expressed as mean value ± standard deviation for normally distributed data or as median with interquartile range for non-normally distributed data. The distribution of variables was evaluated by the Shapiro–Wilk test, the quantile–quantile graph, and the Cullen–Frey graph. The homogeneity of variances was assessed by the Levene test. For comparison of interval data, Student’s t-test for independent samples or the Mann–Whitney U test was used, depending on the data distribution. Categorical variables were compared using the χ^2^ test or Fisher’s exact test. Kaplan–Meier estimates were used to assess the patients’ as well as kidney graft survival (including death censored) according to groups SPK and KTx. Differences between survival curves were assessed using the log-rank test. The analysis of eGFR levels through time in both groups was performed using mixed models for repeated measures (package ‘*mmrm*’) [15]. A compound symmetry covariance matrix was used in the mixed model for repeated measurement analysis, and time was parametrized as a categorical variable. Survival analyses were performed using univariable and multivariable Cox proportional hazards regression models. All variables that were significant in univariable Cox analysis were included in the baseline model for multivariable analysis. The best multivariable model was presented. The proportionality assumption was tested based on the Schoenfeld residuals (function ‘*cox.zph*’). Multicollinearity was assessed based on the correlation matrix of model coefficients and variance inflation factors (VIF). Results were presented as hazard ratios with 95% confidence intervals (CI) and corresponding *p*-values. Mean values of eGFR levels were plotted through follow-up, separately for SPK and KTx groups using the fractional polynomial method [16].

## 3. Results

Patients undergoing KTx alone were older, had higher BMI compared with SPK recipients, and suffered over twice as often from coronary disease. Notably, the duration of T1D prior to transplantation was similar in both groups, and differences in dialysis duration were not statistically significant (Table 1). Regarding donor parameters, SPK recipients received organs from younger donors with lower BMI values. Histocompatibility in terms of both HLA I and II was better in KTx recipients, and shorter cold ischemia time (CIT) in SPK recipients was not associated with kidney graft function in the early postoperative period. SPK recipients more often received induction therapy and were predominantly treated with tacrolimus and mycophenolate acids, whereas in the KTx group, half of the recipients received cyclosporine A, and a significant part received azathioprine.

Simultaneous pancreas transplantation reduced total diabetes duration over the entire follow-up period (Table 2). In patients after KTx alone, myocardial infarction and limb amputation during a follow-up period of up to 20 years were observed significantly more often than in SPK recipients. In the case of kidney graft loss, the rate of kidney retransplantation was higher in the SPK group.

Comparative analyses using two statistical approaches showed no significant differences between the groups in recipient survival, kidney graft survival, and death-censored kidney graft survival, both at five-year intervals and over the entire follow-up period (Table S1, Figure 1). Notably, 10 years after the SPK, over 40% of recipients did not require insulin administration (Table S1, Figure 2).

**Figure 3:**
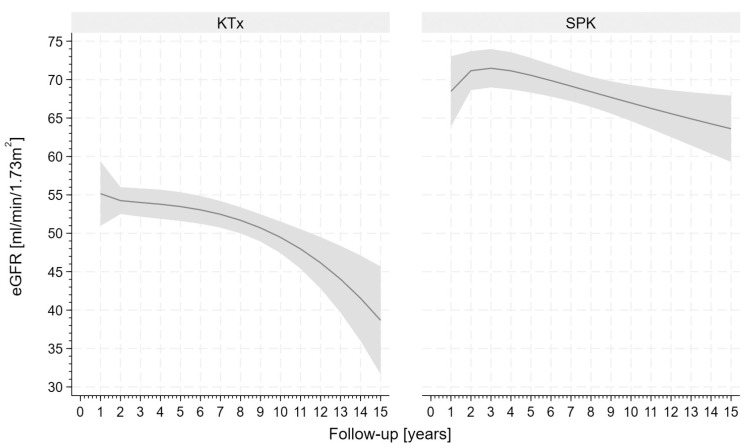
Comparison of estimated glomerular filtration rate (eGFR) between patients after kidney transplantation alone (KTx) and simultaneous pancreas–kidney transplantation (SPK) in a 15-year follow-up period. The gray area presents 95% confidence interval.

The function of kidney graft was superior in SPK recipients compared with kidney-only recipients over the entire observation period (Table S2). Furthermore, in SPK recipients, the estimated glomerular filtration rate was stable compared to the value obtained 1 year after transplantation, whereas in the KTx group, these values were significantly lower (Figure 3). Group (*p* < 0.001) and time (*p* < 0.001) had a significant influence on eGFR values. Moreover, a significant interaction between group and time was observed (*p* < 0.001). In the SPK group, no significant changes through time were observed, while in the KTx group, eGFR values decreased significantly at 5 (*p* < 0.05), 10 (*p* < 0.001), and 15 (*p* < 0.001) years. Proteinuria during the entire follow-up period was observed more frequently in KTx recipients (40.0%) than in SPK recipients (17.9%, *p* < 0.01).

Univariable regression analysis identified several factors influencing recipient, kidney graft, and death-censored kidney graft survival in the entire group of 194 diabetic recipients over a 20-year follow-up period after KTx or SPK (Table S3).

Multivariable regression analysis confirmed that recipient age and duration of dialysis therapy were associated with recipient survival, whereas duration of dialysis therapy, prior cardiovascular events, high sensitization, and episodes of acute rejection were associated with kidney graft survival. Death-censored kidney graft survival was associated with duration of dialysis, high sensitization, HLA class II mismatch, acute rejection episodes, and the presence of proteinuria (Table 3).

## 4. Discussion

The most important finding of this study is the fact that in T1D patients with ESKD who underwent deceased donor transplantation (KTx), the simultaneous transplant of a pancreas did not improve recipient or kidney graft survival compared with kidney-only recipients in the long-term follow-up period after transplantation.

This lack of improvement in survival was observed despite a reduction in total diabetes duration, a lower incidence of cardiovascular episodes (particularly myocardial infarction and limb amputations) after transplantation, as well as better kidney graft function in SPK recipients. Notably, none of these factors had a beneficial influence on recipient or kidney graft survival in the analyzed follow-up period.

When comparing the results of our analysis with those of other European comparative studies, attention should be paid to the differences in clinical parameters between ESKD patients with T1D for whom SPK and KTx were considered, particularly with regard to their age. In our analysis, the mean age of patients qualified for SPK was only 38 years, while in other transplant centers, it ranged from 40.6 to 49.1 years [17,18,19]. The mean age of patients who qualified for KTx only was 43.6 years, whereas in other studies, it ranged from 50 to 55.1 years.

These data suggest that, given the age of patients qualified for KTx alone in Poland, SPK would have also been considered for them in other centers. Furthermore, other centers considered significantly older patients for KTx alone, which may have contributed to their poorer survival after transplantation compared to patients who underwent SPK or KTx alone in our analysis [17,18,19].

The results of our analysis of T1D kidney recipients’ survival can be related to the analysis of the Dutch nationwide cohort conducted by a Leiden team [17]. In this study, Esmeijer et al. revealed that, in patients with T1D and ESKD, a treatment strategy favoring SPK over KTx alone was associated with 44% and 31% reductions in 10- and 20-year all-cause mortality, respectively [17]. Similarly, Lange et al. found that the risk factor-adjusted hazard ratio for mortality in SPK recipients was 0.63 compared to KTx recipients alone [18]. Furthermore, Lindahl et al. demonstrated that 67% of patients survived 10 years after SPK and 36% after KTx [19]. In Sucher et al.’s study, 10-year recipient survival was over twice as high in the SPK group (82%) compared to the KTx group (40%) [20].

According to Polish data published by Czerwinski et al., based on the Polish Organ Transplantation Registry, 10-year recipient survival after SPK is 69%. However, there are no data available on the survival of kidney-only recipients in T1D patients separately [21].

The lack of differences in survival between SPK and KTx recipients may be explained by the high mortality rate observed among SPK recipients due to complications related to pancreas transplantation during the first six postoperative months, particularly in the early phase of the SPK program [22]. It is important to note that early patient mortality after SPK transplantation decreased from 10.9% between 2004 and 2011 to 4.2% between 2012 and 2019. Similar results were observed by other researchers [19]. According to the nationwide Polish registry, the one-year mortality rate of SPK recipients varies from 9 to 15% [21] depending on the center. The comparable long-term survival rates of T1D recipients of SPK and KTx alone may also be influenced by the high survival rate of diabetic patients undergoing KTx alone observed in our cohort, which was 64% after 10 years of follow-up. During the same period after KTx, other authors reported recipient survival rates between 36% and 52% [18,19,20].

As with recipient survival, other authors often observed higher survival rates for kidney graft in SPK recipients compared to KTx recipients. In Lange et al.’s study, the 10-year kidney graft survival rate was 68.5% in the SPK group, which differed significantly from the 27% rate in the KTx alone group [18]. Lindahl et al. also reported a 10-year kidney graft survival rate of 57% for the SPK group and a much lower rate of 30% for the deceased donor KTx alone group [19]. In Sucher et al.’s study, the 10-year kidney graft survival was 69% in the SPK group and 26% in KTx group [20]. Compared to the aforementioned studies, kidney graft survival in our analysis was slightly worse in the SPK group but much better in the KTx alone group. The differences between our study and other reports may result, to some extent, from the higher mortality in the KTx group compared to the SPK group observed by other authors.

Our study revealed that the transplanted kidney functioned significantly worse in the KTx group than in the SPK group. Furthermore, kidney function deteriorated faster in recipients treated with insulin. Moreover, the percentage of patients with proteinuria was more than twice as high throughout the entire observation period in the KTx group compared to the SPK group. While we did not attempt to determine the relationship between eGFR values and factors related to the donor, recipient, or applied treatment, considering that deterioration of renal function in patients with DM1 has slowed significantly in recent years due to better disease management [23,24], the observed differences in renal function between the SPK and KTx groups can primarily be explained by the significantly younger age of SPK donors compared to kidney-only donors: Elbadri et al. confirmed that higher donor age was associated with low eGFR five years post-renal transplantation [25]. Furthermore, Tacrolimus and Cyclosporine A have different nephrotoxicity profiles; hence, the difference in immunosuppressive regimens can constitute an additional confounder affecting long-term eGFR differences between the two groups [26].

Throughout the entire observation period, fewer episodes of myocardial infarction and major amputations were observed in the group of patients undergoing SPK compared to kidney-only recipients. Similar differences in the occurrence of cardiovascular events were recently observed by Lange et al. (7% vs. 28%, respectively) [18]. Sucher et al. confirmed a significantly lower incidence of peripheral vascular complications (32%) in SPK patients compared to T1D patients after KTx (69%) [20]. On the other hand, Lehner et al.’s analysis revealed that early pancreas graft loss was not associated with major adverse cerebral or cardiovascular events over 10 years compared to patients with pancreas functioning after 3 months [27]. The observed differences in CVD progression between recipients may be explained by favorable changes in markers of vascular wall damage after successful pancreas transplantation restores normal glucose metabolism [28,29].

Based on the results obtained, we attempted to determine which factors influence long-term outcomes of KTx in T1D patients with ESKD. The attempts revealed that recipient survival is affected by recipient age and dialysis vintage duration. Kidney graft survival is affected, among other factors, by dialysis vintage duration, episodes of acute rejection, and pretransplant patient sensitization.

Contrary to Lindahl et al., we did not manage to prove that SPK was protective against mortality compared with KTx [19]. To some degree, it is consistent with the results of Lehner et al., who proved that pancreas graft loss within 3 months after SPK did not significantly impact patient or kidney graft survival over 10 years of follow-up [27].

In summary, it remains difficult to demonstrate the superiority of SPK as a treatment option for all patients with T1D who require KTx. Comparing SPK and KTx outcomes in patients with T1D will become even more difficult with the introduction of new technologies in diabetes treatment, including continuous glucose monitoring devices and insulin pumps [30,31,32]. For this reason, further comparative studies of SPK and KTx outcomes based on current data and long-term follow-up are needed. Our analysis, which is a continuation and extension of a study that began 10 years ago in two transplant centers in Poland, meets these criteria [11]. The study’s strength lies in its use of a detailed registry of all KTx cases in Katowice, supplemented with information on all subsequent T1D kidney recipients from the center in Wrocław, as well as data obtained from the transplant center providing long-term care for patients after KTx and SPK in Warsaw. This approach eliminated the problem of missing data, frequently reported by researchers analyzing national databases [17]. The study’s limitations are the small number of transplant patients and the lack of information on recipients’ causes of death, including those due to the SARS-CoV-2 pandemic, which could introduce survival bias into the long-term analysis. It is important to note that during the initial pandemic wave in 2020, up to 90% of solid organ recipients with COVID-19 required hospitalization, and a third of these patients required intensive care or mechanical ventilation. The mortality rates reached 20–25% [33].

## 5. Conclusions

In conclusion, the simultaneous transplantation of a pancreas in patients with type 1 diabetes and end-stage kidney disease undergoing deceased donor kidney transplantation does not improve the survival rate of the recipient or the kidney graft compared with kidney-only recipients during a long-term follow-up period, despite the reduction in the total duration of diabetes, lower incidence of cardiovascular episodes (particularly myocardial infarction and amputation of the limbs), and better kidney graft function.

## Figures and Tables

**Table 1:** Baseline characteristics of patients who underwent simultaneous pancreas–kidney transplantation (SPK) and kidney transplantation alone (KTx) (mean ± SD, median, and IQR or frequency).

	SPK (N = 93) 47.9%	KTx (N = 101) 52.1%	*p* SPK vs. KTx
Recipient			
Recipient age [years]	38.3 ± 7.0	43.6 ± 9.7	<0.001
Female gender [N, %]	45 (48.4)	44 (43.6)	0.50
BMI [kg/m^2^]	22.7 ± 2.5	23.7 ± 3.2	<0.05
BMI ≥ 25 [kg/m^2^]; [N, %]	19 (20.6)	30 (30.0)	0.14
Duration of diabetes prior to Tx [years]	25.4 ± 6.4	25.2 ± 8.4	0.83
Duration of dialysis therapy [months]	18 (12; 33)	24 (15; 38)	0.06
Last dialysis modality [HD] [N, %]	77 (82.8)	77 (77.0)	0.32
Arterial hypertension [N, %]	76 (81.7)	81 (82.6)	0.87
Previous coronary disease [N, %]	8 (8.7)	20 (20.0)	<0.05
Previous cardiovascular episode * [N, %]	6 (6.5)	10 (10.1)	0.37
Previous limb or toe amputation [N, %]	9 (9.8)	3 (3.0)	0.08
Highly sensitized pts (last PRA > 20%) [N, %]	1 (1.1)	5 (5.2)	0.21
Donor			
Donor age [years]	25.4 ± 6.9	43.3 ± 12.5	<0.001
Female gender [N, %]	35 (37.6)	43 (44.8)	0.32
Trauma as the cause of death [N, %]	47 (51.1)	36 (43.4)	0.31
BMI [kg/m^2^]	23.2 ± 2.5	25.4 ± 3.5	<0.001
BMI ≥ 25 [kg/m^2^] [N, %]	15 (16.1)	38 (45.8)	<0.001
Transplantation procedure			
Mismatch HLA I	2.84 ± 0.94	2.42 ± 1.00	<0.01
Mismatch HLA II	1.49 ± 0.58	0.94 ± 0.62	<0.001
CIT for kidney [hours]	8.9 (7.7; 10.2)	20.4 (16.6; 24.0)	<0.001
Immediate kidney graft function [N, %]	44 (47.3)	37 (38.5)	0.10
Slow kidney graft function [N, %]	34 (36.6)	33 (34.4)	
Delayed kidney graft function [N, %]	10 (10.8)	23 (24.0)	
Primary kidney graft non-function [N, %]	5 (5.4)	3 (3.1)	
Immunosuppressive treatment			
Induction therapy [%]	35 (37.6)	24 (23.8)	<0.05
Calcineurin inhibitor: Cyclosporine A	2 (2.1)	49 (48.5)	<0.001
Calcineurin inhibitor: Tacrolimus	91 (97.8)	52 (51.5)	
Antimetabolic drug: Mycophenolate acid	85 (98.8)	80 (84.2)	<0.001
Antimetabolic drug: Azathioprine	1 (1.2)	15 (15.8)	

BMI—body mass index; HD—hemodialysis; PRA—panel reactive antibodies; HLA—human leukocyte antigen; CIT—cold ischemia time. * Cardiovascular episode: myocardial infarction, stroke, or TIA.

**Table 2:** Selected clinical parameters in patients after simultaneous pancreas–kidney transplantation (SPK) and kidney transplantation alone (KTx) within the entire observation period (mean ± SD, median, and IQR or frequency).

	SPK (N = 93) 47.9%	KTx (N = 101) 52.1%	*p* SPK vs. KTx
Duration of follow-up [years]	10.08 ± 5.20	11.5 ± 5.54	0.07
Total duration of diabetes [years]	28.1 ± 7.4	34.9 ± 11.0	<0.001
Total duration of dialysis therapy [months]	24.0 (13.0; 50.0)	27.0 (15.8; 59.0)	0.22
Myocardial infarction [N, %]	5 (6.8)	18 (20.9)	<0.05
Stroke or TIA [N, %]	3 (4.1)	8 (9.3)	0.20
CABG/PTCA [N, %]	6 (8.2)	10 (11.6)	0.48
Cardiovascular episode * [N, %]	8 (11.0)	24 (27.9)	<0.01
Cardiovascular episode * or CABG/PTCA [N, %]	10 (13.7)	24 (27.9)	<0.05
Limb amputation [N, %]	2 (2.7)	10 (11.6)	<0.05
Toe amputation [N, %]	8 (11.0)	5 (5.9)	0.25
Limb or toe amputation [N, %]	10 (13.7)	14 (16.5)	0.63
Acute rejection episode [N, %]	21 (22.8)	29 (29.6)	0.29
Kidney retransplantation [N, %]	7 (7.5)	1 (1.0)	<0.05

TIA—transient ischemic attack; CABG—coronary artery bypass grafting, PTCA—percutaneous transluminal coronary angioplasty. * Cardiovascular episode: myocardial infarction, stroke, or TIA.

**Table 3:** Factors influencing recipient, kidney graft, and death-censored kidney graft survival in a 20-year follow-up period after kidney transplantation alone or simultaneous pancreas - kidney transplantation in type 1 diabetes patients—multivariable Cox regression analysis.

	Recipient Survival		Kidney Graft Survival		Kidney Graft Survival Censored for Death	
	HR	±95% CI	HR	±95% CI	HR	±95% CI
Recipient						
Recipient age [years]	1.042 ^#^	1.018, 1.066	X	X	X	X
Duration of dialysis therapy [months]	1.008 *	1.001, 1.014	1.008 **	1.002, 1.014	1.013 **	1.004, 1.022
Previous cardiovascular episode	X	X	2.248 *	1.215, 4.157	X	X
Highly sensitized pts (last PRA > 20%)	X	X	4.770 **	1.828, 12.45	6.015 **	2.019, 17.92
Acute rejection episode	X	X	1.865 **	1.256, 2.769	2.156 *	1.165, 3.989
Transplantation procedure						
Mismatch HLA II	X	X	X	X	2.323 **	1.348, 4.005
Proteinuria	X	X	X	X	2.118 *	1.116, 4.017
Maximal VIF	1.02		1.06		1.27	

* *p* < 0.05, ** *p* < 0.01, and ^#^ *p* < 0.001; HR—hazard ratio; CI—confidence interval; X—non-significant in univariable Cox regression analysis (Table S3), removed from the model; PRA—panel reactive antibodies; VIF—variance inflation factor.

## Data Availability

The data presented in this study are available upon request from the corresponding author. The data are not publicly available due to privacy restrictions.

## Supplementary Materials

The following supporting information can be downloaded at https://www.mdpi.com/article/10.3390/jcm15072565/s1, Table S1: Patient and kidney graft survival and kidney graft function in simultaneous pancreas–kidney transplantation (SPK) and kidney transplantation alone (KTx) during the follow-up period. Table S2: Estimated glomerular filtration rate and its relative change in patients with simultaneous pancreas–kidney transplantation (SPK) and kidney transplantation (KTx) alone during the follow-up period. Table S3: Factors influencing recipient, kidney graft, and death-censored kidney graft survival in a 20-year follow-up period after kidney transplantation alone or simultaneous pancreas and kidney transplantation in type 1 diabetes patients—univariable Cox regression analysis.

## Abbreviations

The following abbreviations are used in this manuscript: BMI—body mass index; CABG—coronary artery bypass grafting; CIT—cold ischemia time; CVD—cardiovascular diseases; ESKD—end-stage kidney disease; eGFR—estimated glomerular filtration rate; HD—hemodialysis; HLA—human leukocyte antigen; KTx—kidney transplantation; PRA—panel reactive antibodies; PTCA—percutaneous transluminal coronary angioplasty; SPK—simultaneous pancreas-kidney transplantation; T1D—type 1 diabetes; TIA—transient ischemic attack.
