# Supplementary material for: Comparative Analysis of Kidney and Simultaneous Pancreas–Kidney Transplantation: Long-Term Outcomes in Type 1 Diabetic Patients with End-Stage Kidney Disease
Source: J Clin Med. 2026 Mar 27;15(7):2565. doi: 10.3390/jcm15072565 (PMC13073624; doi:10.3390/jcm15072565)
Supplement: Supplementary file 1 [file jcm-15-02565-s001.zip › Ziaja et al Long term outcomes SPK vs KTx Table S1.pdf]

Table S1. Patient and kidney graft survival and kidney graft function in simultaneous pancreas-kidney transplantation (SPK) and kidney transplantation (KTx) during the follow-up period (N); n – the number of patients in the given observation period.

	SPK		KTx		p
	n	N (%) / 95% CI (%)	n	N (%) / 95% CI (%)	
Patient survival					
1 year	93	85 (91.4) / 83.9–95.6	101	99 (98.0) / 93.1–99.5	0.051
5 year	93	77 (82.8) / 73.9–89.1	101	87 (86.1) / 78.1–91.6	0.66
10 years	73	46 (63.0) / 49.3–70.8	89	57 (64.0) / 53.7 – 73.2	0.99
15 years	53	16 (30.2) / 19.5–43.5	79	29 (36.7) / 26.9–47.7	0.56
Kidney graft survival					
1 year	93	80 (86.0) / 77.5–91.6	101	90 (89.1) / 81.5–93.8	0.66
5 year	93	68 (73.1) / 63.3–81.1	101	79 (78.2) / 69.2–85.2	0.41
10 years	73	38 (52.1) / 40.8–63.1	89	49 (55.1) / 44.7–65.0	0.70
15 years	53	12 (22.6) / 13.4–35.5	79	23 (29.1) / 20.2–39.9	0.41
Kidney graft survival censored for death					
1 year	85	80 (94.1) / 87.0–97.5	99	90 (90.9) / 83.6–95.1	0.41
5 year	77	68 (89.6) / 79.2–93.7	87	79 (90.8) / 82.9–95.3	0.78
10 years	46	38 (82.6) / 69.3–90.9	57	49 (86.0) / 74.7–92.7	0.64
15 years	16	12 (75.0) / 50.5 – 89.8	29	23 (79.3) / 61.6–90.1	1.00
Pancreas graft survival					
1 year	93	71 (76.4) / 66.8–83.8	-	-	-
5 year	93	55 (59.1) / 49.0–68.6	-	-	-
10 years	73	32 (43.8) / 33.1–55.3	-	-	-
15 years	53	8 (15.1) / 7.8–27.1	-	-	-
Pancreas graft survival censored for death					
1 year	85	71 (83.5) / 74.2–89.9	-	-	-
5 year	77	55 (71.4) / 60.5–80.3	-	-	-
10 years	46	32 (69.6) / 55.2–80.9	-	-	-
15 years	16	8 (50.0) / 28.0–72.0	-	-	-

CI – 95% confidence interaval
