# Supplementary material for: Comparative Analysis of Kidney and Simultaneous Pancreas–Kidney Transplantation: Long-Term Outcomes in Type 1 Diabetic Patients with End-Stage Kidney Disease
Source: J Clin Med. 2026 Mar 27;15(7):2565. doi: 10.3390/jcm15072565 (PMC13073624; doi:10.3390/jcm15072565)
Supplement: Supplementary file 1 [file jcm-15-02565-s001.zip › Ziaja et al Long term outcomes SPK vs KTx Table S2.pdf]

Table S2. Estimated glomerular filtration rate and its relative change in patients with simultaneous pancreas-kidney transplantation (SPK) and kidney transplantation (KTx) alone during the follow-up period.

| | SPK | KTx | $p_{SPK\ vs\ KTX}$ |
|---|---|---|---|
| | N/ median / IQR | N/ median / IQR | |
| 1 year | 81/ 70.5 / 24.0 | 89/ 54.8 / 23.0 | < 0.001 |
| 5 year | 62/ 70.8 / 28.1 | 73/ 49.3 / 23.2 | < 0.001 |
| $\Delta_{\%}$ 5 years vs 1 year | 62/ 3.76 / 30.3 | 73/ -5.72 / 17.0* | < 0.01 |
| 10 years | 41/ 65.9 / 22.9 | 41/ 47.1 / 18.3 | < 0.001 |
| $\Delta_{\%}$ 10 years vs 1 year | 41/ -6.0 / 34.5 | 41/ -18.6 / 34.7* | < 0.05 |
| 15 years | 14/ 68.1 / 27.5 | 11/ 35.4 / 20.8 | < 0.001 |
| $\Delta_{\%}$ 15 years vs 1 year | 14/ 11.7 / 35.6 | 11/ -43.8 / 31.9* | < 0.05 |

* $p < 0.001$ – comparison between the two compared periods
