# Supplementary material for: Comparative Analysis of Kidney and Simultaneous Pancreas–Kidney Transplantation: Long-Term Outcomes in Type 1 Diabetic Patients with End-Stage Kidney Disease
Source: J Clin Med. 2026 Mar 27;15(7):2565. doi: 10.3390/jcm15072565 (PMC13073624; doi:10.3390/jcm15072565)
Supplement: Supplementary file 1 [file jcm-15-02565-s001.zip › Ziaja et al Long term outcomes SPK vs KTx Table S3.pdf]

Table S3. Factors influencing recipient, kidney graft, and death censored kidney graft survival in a 20-year follow-up period after kidney or simultaneous pancreas-kidney transplantation in type 1 diabetic patients – univariable Cox regression analysis.

	Recipient survival		Kidney graft survival		Kidney graft survival censored for death	
	HR	± 95% CI	HR	± 95% CI	HR	± 95% CI
SKP vs KTx	0.919	0.616, 1.372	0.984	0.681, 1.423	0.979	0.580, 1.652
Recipient						
Female gender	0.933	0.631, 1.379	0.988	0.688, 1.418	1.340	0.802, 2.240
Recipient age [years]	1.041#	1.018, 1.064	1.022*	1.001, 1.043	0.983	0.953, 1.014
BMI ≥ 25 [kg/m^2] [N, %]	1.699*	1.108, 2.605	1.395	0.927, 2.102	0.992	0.531, 1.852
Duration of diabetes prior to Tx [years]	1.004	0.976, 1.033	1.000	0.974, 1.026	0.980	0.944, 1.018
Duration of dialysis therapy [months]	1.006$	1.000, 1.012	1.008*	1.002, 1.015	1.009*	1.001, 1.017
Previous cardiovascular episode	2.208**	1.221, 3.992	1.966*	1.095, 3.528	1.389	0.551, 3.503
Highly sensitized pts (last PRA>20%) [N, %]	1.202	0.440, 3.286	4.151#	1.790, 9.627	4.723#	1.671, 13.35
Acute rejection episode	1.517*	1.004, 2.292	1.988#	1.352, 2.922	2.242**	1.314, 3.826
Donor						
Female gender	0.933	0.627, 1.388	1.011	0.699, 1.464	1.142	0.676, 1.930
Donor age [years]	1.009	0.995, 1.024	1.014*	1.001, 1.028	1.018	0.998, 1.038
Trauma as the cause of death	0.864	0.567, 1.316	0.771	0.521, 1.139	0.865	0.503, 1.487
BMI ≥ 25 [kg/m^2]	1.158	0.742, 1.807	0.961	0.630, 1.467	0.827	0.441, 1.553
Transplantation procedure						
Mismatch HLA I	0.929	0.765, 1.127	0.978	0.817, 1.172	0.972	0.752, 1.255
Mismatch HLA II	1.114	0.808, 1.536	1.228	0.920, 1.638	1.502$	0.994, 2.271
CIT for kidney [hours]	1.003	0.979, 1.027	1.004	0.982, 1.027	1.002	0.970, 1.035
Immediate kidney graft function [N, %]	Ref.		Ref.		Ref.	
Slow kidney graft function [N, %]	1.477	0.926, 2.355	1.150	0.746, 1.771	1.203	0.636, 2.276
Delayed kidney graft function [N, %]	1.468	0.861, 2.504	1.464	0.892, 2.404	1.732	0.861, 3.483
Immunosuppressive treatment						
Induction therapy [%]	0.975	0.582, 1.634	1.186	0.751, 1.874	1.319	0.712, 2.442
Proteinuria* [N, %]	0.912	0.578, 1.439	1.362	0.907, 2.045	1.914*	1.091, 3.356

$ $p < 0.1$, * $p < 0.05$, ** $p < 0.01$, # $p < 0.001$; HR – Hazard Ratio; CI – Confidence Interval
